# Mental disorders as risk factors: assessing the evidence for the Global Burden of Disease Study

**DOI:** 10.1186/1741-7015-9-134

**Published:** 2011-12-16

**Authors:** Amanda J Baxter, Fiona J Charlson, Adele J Somerville, Harvey A Whiteford

**Affiliations:** 1 Queensland Centre for Mental Health Research, The Park - Centre for Mental Health, Wacol, Qld 4076, Australia; 2 School of Population Health, University of Queensland, Herston Road, Herston, Qld 4006, Australia

## Abstract

**Background:**

Mental disorders are associated with a considerable burden of disease as well as being risk factors for other health outcomes. The new Global Burden of Disease (GBD) Study will make estimates for both the disability and mortality directly associated with mental disorders, as well as the burden attributable to other health outcomes. Herein we discuss the process by which health outcomes in which mental disorders are risk factors are selected for inclusion in the GBD Study. We make suggestions for future research to strengthen the body of evidence for mental disorders as risk factors.

**Methods:**

We identified a list of potential associations between mental disorders and subsequent health outcomes based on a review of the literature and consultation with mental health experts. A two-stage filter was applied to identify mental disorders and health outcomes that meet the criteria for inclusion in the GBD Study. Major limitations in the current literature are discussed and illustrated with examples identified during our review.

**Results and discussion:**

Only two associations are included in the new GBD Study. These associations are the increased risk of ischemic heart disease with major depression and mental disorders as a risk factor for suicide. There is evidence that mental disorders are independent risk factors for cardiovascular disease (CVD), type 2 diabetes and injuries. However, these associations were unable to be included because of insufficient data. The most common reasons for exclusion were inconsistent identification of 'cases', uncertain validity of health outcomes, lack of generalizability, insufficient control for confounding factors and lack of evidence for temporality.

**Conclusions:**

CVD, type 2 diabetes and injury are important public health policy areas. Prospective community studies of outcomes in patients with mental disorders are required, and their design must address a range of confounding factors.

## Background

New Global Burden of Disease (GBD) Study estimates due for release in 2012 aim to reflect the impact of morbidity and mortality for all diseases, injuries and risk factors, including mental disorders [1]. Although disorder-specific morbidity and mortality are needed for GBD estimates and to guide health policy in the provision of treatment services, quantifying the additional burden due to health outcomes for which those disorders are risk factors provides an additional evidence base for developing preventative health policy [2]. The Comparative Risk Assessment (CRA) component within the GBD Study will be used to evaluate the impact of risk factors on public health within a unified framework [2].

Risk factors are defined within the CRA as exposures that increase the probability of disease or injury [3]. Considered in the CRA are risk factors likely to be among the leading causes of disease burden at a global or regional level [2]. Other inclusion criteria require that risk factors be sufficiently well-defined, such that population distribution can be quantified and should comprise those for whom the exposure is modifiable [2]. CRA requirements specify that data should be available to quantify the risk of health outcomes for given exposures and sufficient strength of evidence for causality based on collective scientific knowledge [2].

In considering strength of evidence for risk factors in health outcomes, the Bradford-Hill criteria are widely accepted as a guide for assessing the evidence base [4,5]. The Bradford-Hill criteria describe the best evidence as that which demonstrates the strength and consistency of the relationship, the specificity of the risk factor and outcome, a temporal sequence in which the risk precedes the outcome, and experimental evidence such as the effect of preventative action [4].

Although previous GBD studies included outcomes for illicit drug use and alcohol use [6], the current GBD Study is the first in which other mental disorders are considered as independent risk factors for other health outcomes. A growing body of literature reports links between mental disorders and other health conditions. Higher rates of morbidity and mortality have been reported in psychiatric patients for decades. Longitudinal studies have demonstrated links between mental disorders and cardiovascular disease [7], metabolic disease [8] and injury [9].

Our aim in this paper was not to present the results of a series of systematic reviews of the strength of evidence for health outcomes. To review the evidence comprehensively, each potential outcome for mental disorders deserves in-depth discussion beyond the scope of this paper. Rather, this paper describes the process by which health outcomes were selected for inclusion in the GBD 2010 Study. We identify the most common reasons that potential outcomes were not included and provide suggestions for future research. The GBD CRA component for illicit drug use and alcohol use is outside the scope of this paper and will be reported separately by the groups undertaking that work.

## Methods

We reviewed the current literature for data on the strength of evidence for mental disorders as risk factors for other health outcomes. Mental disorders included in the GBD 2010 Study are anxiety disorders, depressive disorders (major depression and dysthymia), bipolar disorder, schizophrenia, eating disorders, autistic spectrum disorders, conduct disorder and attention-deficit/hyperactivity disorder (ADHD). Patients with these disorders are defined as those meeting the diagnostic criteria for either the *ICD-10 Classification of Mental and Behavioural Disorders: Diagnostic Criteria for Research *(ICD-10) classification system [10] or the *Diagnostic and Statistical Manual of Mental Disorders, Fourth Edition-Text Revision *(DSM-IV-TR) [11]. The disorders should not be substance-induced or due to a general medical condition, as these are covered under other categories within the GBD 2010 Study.

Potential health outcomes were identified using a consultative heuristic approach. Good-quality, peer-reviewed evaluations of the literature were initially sought. A review by Prince and colleagues [12] provided the basis for subsequent investigation. Outcome-specific reviews were then identified to examine the breadth of data available to determine the strength of the evidence. We consulted mental health epidemiologists and clinicians with a special interest in the field, and their suggestions were included. The Mental Disorders and Illicit Drug Use Expert Group was then invited to provide feedback on the proposed outcomes and to contribute additional suggestions.

A two-stage filter was then applied to identify those associations that met criteria for inclusion based on the parameters of the GBD 2010 Study and the specific requirements for risk factors and outcomes of the CRA project. The outcomes were first filtered by the GBD Study requirement that the resulting health condition must fall within the study classifications of disease and injury. The second filter focused on the CRA requirements. These criteria included clear definition of risks and outcomes and sufficient strength of evidence for an independent association guided by the Bradford-Hill criteria. GBD Study estimates aim to reflect loss of health. The methodology excludes 'out of skin' elements of functioning, such as participation restrictions which, though important, are beyond the scope of what is captured by disability-adjusted life years [13,14]. Therefore, outcomes such as social consequences are beyond the scope of this study.

## Results

A number of good-quality reviews of health outcomes for patients with mental disorders were identified [7-9,12,15]. These reviews informed the initial list of health outcomes. Six health outcomes were considered after application of the first filter (Table 1). Examples of associations that did not meet GBD Study criteria were those with non-health-related outcomes, such as increased risk of unemployment arising from depression [16].

**Table 1:** Health outcomes for mental disorders considered for inclusion in the Comparative Risk Assessment component within the Global Burden of Disease study*

Mental disorder as risk factors	Health outcomes
Depression, bipolar disorder and anxiety disorders	CHD
Depression and anxiety disorders	CVD
Depression, bipolar disorder and schizophrenia	Type 2 diabetes
Pervasive developmental disorders	Injury
Childhood behavioural disorders	Injury
Mental disorders	Suicide

CHD = coronary heart disease; CVD = cardiovascular disease. *Following application of the first filter: Global Burden of Disease 2010 requirements for inclusion.

After applying the second filter, sufficient evidence was identified to include attributable burden for two health outcomes: suicide and ischemic heart disease [17]. A report of data identified for major depression, schizophrenia, bipolar disorder, anxiety disorders and anorexia nervosa as risk factors for suicide is currently in preparation and will be submitted for peer review (unpublished data: A. Ferrari). Given that only two conditions were included in the CRA component of the GBD 2010 Study, a reasonable question is why the attributable burden of other health outcomes is not reflected in the estimates. To illustrate the nature of the limitations in the current literature, examples are provided for associations that are likely to have a substantial impact on the global burden of disease.

## Discussion

The most significant factors that limited inclusion of studies and ultimately prevented inclusion of other health outcomes in the new GBD Study estimates are (1) risk factor case identification, (2) health outcome case identification, (3) generalizability, (4) confounding factors and (5) temporality.

### Risk factor case identification

To attribute the proportionate burden of a condition, both risks and outcomes must be clearly defined [18]. For inclusion in the GBD CRA component, mental disorders considered as risk factors must meet the DSM-IV-TR or ICD-10 diagnostic criteria, which require a threshold level of symptomatology and, in some cases, impairment. The current strength of evidence for disorders such as anxiety disorders and childhood behavioural disorders as risk factors is hampered by the lack of consistency in the definitions used to determine the presence of these disorders.

#### Inconsistent definitions

In our review, we found that the definitions used when mental disorders were considered as risk factors varied considerably. The most common issue identified was the use of broad definitions, such as the use of 'anxiety' but its' being unclear with regard to whether this term referred to an anxiety disorder that met diagnostic criteria. Some studies tend to describe anxiety as being present on the basis of dimensional scales. These scales (for example, brief symptom checklists, lists of personality traits or measures of psychological distress) can be administered quickly and demonstrate good sensitivity. However, their low specificity [19] results in the inclusion of cases of anxiety that do not reach the threshold for a diagnosis. The issue is highlighted by a meta-analytic review of 21 studies exploring anxiety constructs and incident CHD and cardiac mortality [7]. Only two studies that reported a significant association defined 'anxiety' according to DSM-IV-TR and/or ICD-10 diagnostic criteria [7]. Whilst acknowledging that symptoms of mental disorders occur along a continuum, a threshold must be employed to derive comparable estimates of attributable burden. The question whether psychological distress and anxiety symptoms increase the risk for CHD is valid, but it does not directly allow for estimation of the extent to which one disorder (in this case, an anxiety disorder) is a risk factor for another disorder (CHD).

#### Incomplete identification of cases

Cross-sectional studies rely on retrospective data collection to establish patients' mental health history. These studies need to be interpreted with caution, as the accuracy of recall of symptoms of mental disorders is unreliable [20]. To illustrate, clinically defined ADHD requires the onset of symptoms and impairment before age seven years [11]. Based on recall, the onset for externalizing disorders such as ADHD, conduct disorder (CD) and oppositional defiant disorder (ODD) is generally estimated to be later than actual onset [21], resulting in potential misclassification of cases as noncases. Higher rates of accidental injury are reported for people with behavioural disorders such as ADHD [22,23]; however, recall bias increases the chance that cases of ADHD are treated as noncases. In trying to quantify the increased risk due to mental disorders, variably defined 'cases' limit the ability to infer a consistent relationship because the characteristics of people with the risk factor are likely to be inconsistent between studies.

### Health outcome case identification

The CRA framework requires that attributable burden be associated with a specific health condition [2]. A number of proposed outcomes could not be included because of the reliability of studies on self-reported measures (for example, type 2 diabetes) or on outcomes not specific to a health condition, such as vehicular accidents.

#### Health outcomes that cannot be verified

Biomedical measures are rarely collected in community-based studies, relying instead on self-report of health outcomes. The accuracy of self-report measures depends on the community rate of diagnosis and participants' recall and knowledge. Chronic diseases such as type 2 diabetes are particularly difficult to identify on the basis of survey data. In developed countries, almost 50% of type 2 diabetes cases are undiagnosed [24]. In a sample of patients with clinically diagnosed diabetes, only two of three patients self-reported the presence of diabetes [25]. Systematic underreporting of the outcome may underestimate or obscure the association between risk and outcome.

#### Outcomes that cannot be mapped to a health condition

An additional issue related to outcomes is the reporting of events (for example, vehicle collisions) rather than the health implication of the event (for example, injuries). For example, two meta-analytic reviews found strong evidence for an association between ADHD and negative driving injury outcomes, including poor performance on simulators, traffic violations and motor vehicle collisions [9,26]. A causal pathway is plausible, considering that common ADHD characteristics include inattention, distractibility, impulsiveness and slow processing ability [9]. However, the usability of findings is limited in the attribution of disease burden because of the lack of information regarding specific health outcomes.

### Generalizability of the sample

Prospective longitudinal studies of outcomes in representative community cases of mental disorders are lacking, especially for severe mental disorders such as bipolar disorders and schizophrenia. As estimates of attributable burden in the GBD CRA component are applied at the population level, studies with samples not sufficiently representative of the general population could not be included. For example, a number of well-conducted studies examining bipolar disorder [27-29] and schizophrenic disorders [27] as risk factors for chronic disease focused on clinical samples. Hospitalized patients have more complex psychopathology and psychological-physical comorbidity that lead to greater risk of negative health outcomes. The degree of association may be overestimated if only severe cases are included.

Clinical samples are subject to a number of biases which may not be controlled for. Clinically ascertained samples are likely to have received treatment, with treatment rates generally being lower in community samples. Because of their use of health services, psychiatric patients with independent risk factors for physical disorders (for example, hypertension) are more likely to have these risk factors recognized and treated compared to community cases. Such sources of systemic bias hamper interpretation of studies based on clinical samples. This limits the generalizability of the risk for health outcomes at the population level.

### Confounding factors

An important issue in establishing the strength of evidence is whether the association persists after adjustment for confounding factors. A confounder is a variable that can cause or prevent the outcome and is also associated with the risk factor [30]. This may lead to a false-positive that erroneously suggests a causal relationship between the dependent variable and the outcome. Type 2 diabetes and CHD are examples of outcomes for which we found the strength of the evidence was further limited because of inability to control for multiple risk factors.

Causal pathways of chronic disease and injury are generally multifactorial. For example, the INTERHEART Study identified nine potentially modifiable risk factors for acute myocardial infarction, including lipid abnormality, hypertension, diabetes, abdominal obesity, consumption of fruits and vegetables, smoking, alcohol intake, physical activity and psychosocial factors [31]. Mental disorders are independently associated with many of these risk factors [32]. To clarify the relationship between mental disorders and CHD, analyses need to control for potentially confounding effects, given the network of factors involved in the development of CHD.

#### Adjustment for comorbidity

A review of the literature [7] found that the majority of studies that examined anxiety disorders and CHD controlled for smoking and physiological factors, yet only two studies reported adjustment for depressive symptoms and none adjusted for the impact of other comorbid mental disorders. Anxiety disorders frequently co-occur with other mental disorders, such as mood disorders and substance abuse [33], which may have a synergistic effect on the development of chronic health conditions [34].

The confounding factors that can be considered in a study vary considerably, due in part to limitations such as cost and burden on respondents. Moreover, insufficient numbers of patients meeting the criteria for the confounder, such as comorbid mental disorders, reduce researchers' capacity to detect an effect. For example, a long-term prospective study of the risk of CHD in community cases of anxiety disorder found that a significant association remained after controlling for a range of confounders [32], but the investigators were unable to control for comorbid depression. The reason for this was that the low number patients with comorbid depression and anxiety prevented adjustment for potentially additive effects of this comorbidity (personal correspondence{ I. Janszky, 2011 }).

#### Effects of treatment

Treatment of a mental disorder affects the course of the disorder and also introduces the risk of iatrogenic outcomes, such as glucose intolerance or atherosclerosis. Reliable data on treatment regimen, including medications, are rarely captured or controlled for in community studies. Researchers in prospective studies of depression and CVD, for example, either do not collect treatment data [35] or collect incomplete information [36].

Treatment may increase the risk of negative health outcomes in some cases or may provide a protective effect in others. Antipsychotic medications have a well-known potential to increase the risk of obesity and abnormal glucose metabolism [15]. Observational studies of women have found that antidepressant use is associated with an increased risk of sudden cardiac death [37] and stroke [38]. Conversely, it has been postulated that the reduction in the duration of psychopathology and symptom severity due to treatment may reduce the risk for chronic physical disease. A potential mechanism suggested by animal studies is that antidepressants may inhibit the expression of proinflammatory cytokines implicated in the progression of CVD [39]. To clarify the multidirectional impact of treatment on health outcomes, researchers need to consider the interactions between treatment type, symptom severity and potential comorbidities.

#### Environmental factors

The current literature suggests a strong link between childhood psychopathology, such as autistic disorders and behavioural disorders, and increased risks for accidental and self-inflicted injuries [40-42]. However, inclusion for CRA is hindered by unrepresentative sampling (registry data or small sample sizes) and lack of data on potential comorbid and environmental risk factors. Common comorbid conditions, including epilepsy and intellectual disability [43], and environmental factors, such as the family context and unsafe physical environments [42], may inflate the risk of injury. Obtaining objective data on family and physical environments is difficult and is rarely collected when children with injuries present for treatment.

### Temporality

Determining temporality and establishing which condition precedes the other constitute the greatest areas of contention in establishing an argument for mental disorders as a risk factor. Temporality of onset is subject to bias arising from self-report of onset and from the use of routinely collected clinical data. Prospective cohort studies, whilst still subject to some degree of bias, produce the best empirical evidence for temporality in risk factor associations.

#### Clinical data

Bias can arise from medical records, which document the diagnosis and the start of treatment more accurately than the onset of the disorder. Symptoms of mental disorders and chronic physical health conditions are often present for some time prior to clinical diagnosis. For example, studies based on data linkage of medical records have reported higher rates of type 2 diabetes in people diagnosed with a mental disorder [27,44]. When the diagnosis of either a mental disorder or diabetes precedes the other, the one diagnosed first may be a risk factor for the other. However, temporal pathways are complicated by the lag time between the onset of the disorder and diagnosis. The high rate of undiagnosed diabetes [24] and the delay between symptom onset and diagnosis of mental disorders confounds temporality.

#### Recall bias

Because of recall bias, inaccurate estimates of the occurrence of disorders can arise from retrospectively collected survey data. For instance, brain injury can induce neurobehavioral changes. However, the subsequent effects can be delayed, as the more significant damage tends to ensue from a series of interrelated processes that affect the central nervous system [45]. If the injury leads to cognitive or behavioural changes, incorrect recall of the onset of occurrence might lead to the assumption that childhood disorders are a risk factor rather than an outcome.

### Future directions for research

Recommendations for future research required to address gaps in the current body of evidence can be generically applied across the majority of risk factors and related health outcomes. Prospective studies of representative community samples using clear and consistent definitions of the mental disorder and outcome are essential to providing evidence for risk. Confounding factors need to be considered, including identifying and controlling for comorbid physical and mental disorders. Specific recommendations are summarized in Table 2.

**Table 2:** Suggested directions for future research

Risk factors and outcomes	Future directions for research
Anxiety disorders as risk factors for CHD and stroke	Population-based prospective studies are needed that clearly define both anxiety disorders and the outcome of interest according to internationally accepted diagnostic criteria. Screening for undiagnosed or subthreshold CVD at baseline to determine the direction of temporal association is needed. Possible confounders, including depression, should be identified, measured and controlled for in statistical models. Determine whether the risk for CVD is reduced in people who receive an evidence-based intervention for anxiety.
Bipolar disorder and schizophrenia as risk factors for CHD, stroke and type 2 diabetes	Prospective studies of representative community samples are needed to reduce the risk of recall bias and temporal ambiguity. The direction of association will be further clarified through baseline screening for undiagnosed or subthreshold chronic disease. In data collection and analysis, the possible bidirectional effects of treatment should be considered in addition to other known risk factors.
Depression as a risk factor for stroke and type 2 diabetes	Longitudinal studies are required where consistent clinical definitions are used to identify patients with depression. Screening for undiagnosed or subthreshold CVD and diabetes at baseline would strengthen the case for temporality. Risk of confounding can be reduced by the collection of data on treatment modality, adherence to treatment and comorbid disorders at both baseline and follow-up. The use of accepted diagnostic criteria should be considered when identifying the potential outcome.
Autistic spectrum disorders and injury	Prospective studies of birth cohorts with frequent follow-up to help determine the order of onset are needed. Where the sample size is limited, multisite collaborations should improve the power of statistical analyses in outcomes of patients with relatively rare childhood disorders. Data collection should encompass possible confounders including common comorbidities (for example, epilepsy, intellectual disability) and environmental factors (for example, level of supervision, safety of physical environment). Data on environmental factors are difficult to obtain, but collaboration between qualitative and quantitative researchers provides scope for novel pathways in data collection on complex issues. Studies of non-Western populations would contribute to the generalizability of findings outside the current narrow social context.
Childhood behavioural disorders and injury	Prospective studies with community samples are needed to establish the order of onset for health outcomes. An approach that has been underutilized thus far is the long-term follow-up of cases from national childhood mental health surveys. Adjustment should be made for possible confounders such as common comorbidities (for example, conduct disorder, substance use) and treatment. Consideration should be given to extending the collection of data beyond vehicular accidents to capture the proportion of people who suffer an injury, as well as the type and severity of injury.

CHD = coronary heart disease; CVD = cardiovascular disease.

## Summary

Whilst preliminary evidence suggests that mental disorders are linked to a range of poor health outcomes, further research is needed to confirm the strength of these relationships. The criteria discussed in this paper may be useful in the design of future studies. CVD, diabetes and accidental injury are major areas of concern for public health policy. If mental disorders are indeed shown to be independent risk factors for these outcomes, accurate diagnosis and treatment of mental disorders may lead to a reduction in the global burden of important health outcomes. As more consistent and compelling evidence becomes available, it is anticipated that these health outcomes will be considered for inclusion in future burden-of-disease studies.

## Competing interests

The authors declare that they have no competing interests.

## Authors' contributions

AB made a substantial contribution to the conception and design of the paper, played a principal role in drafting the article and revising it critically for important intellectual content, performed the final critical editing and approved the version of the manuscript to be published. FC and AS made substantial contributions to the acquisition and interpretation of data, played an important role in drafting the manuscript and revising it critically for important intellectual content, performed the final critical editing and approved the version of the manuscript to be published. HW played an important role in drafting the article, revised it critically for important intellectual content, performed final critical editing and approved the version of the manuscript to be published. All authors read and approved the final manuscript.

## Pre-publication history

The pre-publication history for this paper can be accessed here:

http://www.biomedcentral.com/1741-7015/9/134/prepub
